# Reversible control of current across lipid membranes by local heating

**DOI:** 10.1038/srep22686

**Published:** 2016-03-04

**Authors:** Patrick Urban, Silke R. Kirchner, Christian Mühlbauer, Theobald Lohmüller, Jochen Feldmann

**Affiliations:** 1 Photonics and Optoelectronics Group, Department of Physics and Center for NanoScience (CeNS), LMU Munich, Amalienstraße 54, Munich, 80799, Germany; 2 Nanosystems Initiative Munich (NIM), Schellingstraße 4, 80539 Munich, Germany

## Abstract

Lipid membranes are almost impermeable for charged molecules and ions that can pass the membrane barrier only with the help of specialized transport proteins. Here, we report how temperature manipulation at the nanoscale can be employed to reversibly control the electrical resistance and the amount of current that flows through a bilayer membrane with pA resolution. For this experiment, heating is achieved by irradiating gold nanoparticles that are attached to the bilayer membrane with laser light at their plasmon resonance frequency. We found that controlling the temperature on the nanoscale renders it possible to reproducibly regulate the current across a phospholipid membrane and the membrane of living cells in absence of any ion channels.

The cell membrane is a protective barrier between the cell interior and the external environment, which is almost impermeable for most substances such as drugs, charged molecules and in particular ions^1^. Membrane transport, however, is essential for many vital processes that involve cell signaling^2^ or cell-cell communication^3^ and to establish and regulate electrochemical gradients^4^, osmosis^5^, and intracellular pH levels^6^. Transport from the outside to the inside of a cell or vice versa is usually regulated by specialized membrane channels and transport proteins that can be triggered by chemical ligands^7^, voltage^8^, mechanical activation^9^, or even temperature^10^. Attempts to control the transport mechanism usually involve addressing specific membrane channels by either chemical or physical means. Biochemical approaches were shown to be extremely powerful for controlling cell activity in even living organisms^11^ with light and with high spatio-temporal resolution^12^,^13^,^14^. These methods, however, require the genetic manipulation of target cells^15^ or the chemical synthesis of light-sensitive molecules and drugs^16^.

Alternatively, physical manipulation, such as the absorption of infrared (IR) light alone can already lead to a small temperature rise of the cell membrane which has been shown to be sufficient to excite an action potential in neuronal cells without the requirement of further biochemical modification^17^. This strategy of generating heat with light to control cell function combines the benefits of being non-invasive and universally applicable to any cell type. Heating a cell with a focused laser beam, however, requires relatively high laser powers since light absorption by the thin cell membrane is weak. Furthermore, the large volume of the laser spot leads to a temperature increase of a much larger part of the cell than the surface alone, which usually imposes also a high risk of photodamage.

Plasmonic particles can be applied for a more controlled and efficient way of membrane heating on the nanoscale. Gold nanoparticles absorb light very efficiently at their plasmon resonance frequency^18^. A particle that is attached to a cell, can thus be used to heat an area that can be much smaller than the diffraction limited focus of a laser beam^19^. In recent years, plasmonic heating has been successfully used in manifold applications including plasmon enhanced gene transfection^20^, nanoparticle delivery^21^, and the stimulation of neurons^22^,^23^. Yet, many details about the underlying mechanism that leads to membrane permeability upon localized heating, particularly on a single particle level, are still enigmatic. Temperature, for example, can cause local phase transitions in bilayer membranes with immediate consequences on phospholipid mobility^19^. It has been reported, that fluorescent dyes can leak out of giant unilamellar vesicles made from dipentadecanoylphosphatidylcholine (DC_15_PC) membranes that undergo a gel to fluid transition above 35 °C^24^. Furthermore, it has been discussed that sufficient plasmonic heating of nanorods and nanorod clusters can lead to local membrane rupture and the formation of transient pores^25^,^26^. However, there are some limitations to precisely and reproducibly control membrane permeability based on membrane ‘melting’ or pore formation. First, phase transitions of phospholipid membranes are observed only for certain membrane compositions at physiological temperatures^27^. Second, the formation of pores in cell membranes requires rather strong heating of the plasmonic nanoparticles with temperatures way above the physiological tolerance of cells^21^. Finally, it has been shown that an increase of temperature also leads to a slightly higher mobility of phospholipid molecules^28^. This could already have an immediate effect on the membrane’s electrophysiological properties which has not been investigated to date.

Here, we report that local plasmonic heating of a single gold nanoparticle can be applied to control membrane currents and conductance states of fluid phospholipid membranes without the occurrence of phase transitions or nanopore formation. Optical excitation of spherical, 80 nm gold particles at a frequency close to the surface plasmon resonance results in the generation of heat. We found that the increase in temperature from illuminating a single nanoparticle immediately and fully reversibly affects the conductance of a free standing bilayer membrane. This was observed by recording the changes in membrane current which arises under applied bias by using a planar patch-clamp configuration^25^,^26^,^29^,^30^,^31^,^32^. The amount of current was thereby depending on the laser power and the number of particles that were irradiated at the same time. Finally, we demonstrate how subsequential localized heating can be applied to reversibly control the membrane current of a living cell, even in absence of any temperature sensitive ion channels.

## Results

**Figure 1:**
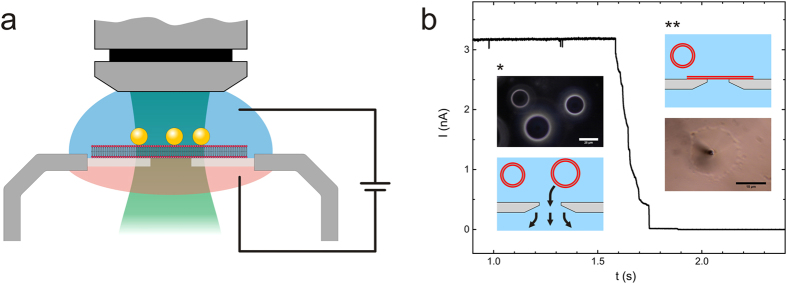
Control of lipid bilayer permeability with heated gold nanoparticles. (**a**) Schematic of the setup. Current measurements are performed with a planar patch clamp system. The lipid bilayer is formed on a glass slide with a small hole in aqueous solution. Gold nanoparticles are deposited on the bilayer. The laser is focused on the sample and the current is monitored with standard bright field microscopy. (**b**) Typical current signature of the formation of a ‘gigaseal’. First, suction is applied to the chip and a GUV (dark field image shown as inset) positions itself randomly on the small aperture (*). When the vesicle ruptures it forms a tight, GΩ seal with the glass around the hole (**schematic and bright field observation).

A schematic of the experiment is shown in Fig. 1a. A bilayer membrane made of diphytanoyl phosphatidylcholine (DPhPC) phospholipid molecules was prepared and formed over the hole in a glass cover slip of a planar patch clamp device. The bilayer was obtained by adsorption and rupture of a giant unilamellar vesicle (for preparation details, please see Materials and Methods). Bilayer membranes made from phospholipid molecules are a well-established model system to mimic the properties of a native cell membrane^33^,^34^,^35^,^36^. The phytanoyl lipids were chosen for membrane preparation as bilayer membranes prepared with these molecules were reported to exhibit good chemical stability and high electrical resistance which renders them particularly suited for electrophysiology measurements^37^. Furthermore, synthetic membranes made of DPhPC have only a very low, but finite permeability for ions due to their hydrophobic interior^38^ and show no phase transition in the temperature range between −120 °C and +120 °C^39^. A small amount of cholesterol was added to the lipid mixture to further increase the stability of the bilayer^40^. Rupture and spreading of lipid vesicles over the hole in the glass slide were observed by the sudden formation of a gigaohm resistance and a current drop by three orders of magnitude compared to the case when the hole in the glass support was not covered (Fig. 1b).

Gold nanoparticles were immersed into the solution and incubated above the bilayer for a few minutes to allow random deposition of statistically at least one nanoparticle on top of the lipid membrane (supporting information Fig. S1). Next, a laser with a wavelength of λ = 532 nm was focused on the sample. The laser wavelength was chosen to be resonant with the particle plasmon to ensure strong light absorption and efficient particle heating. Since the particles on top of the free standing membrane cannot be located under the microscope, the laser spot was chosen to illuminate the entire membrane covering the hole in the glass cover slip. In this configuration, the presence of gold nanoparticles and their successful heating is indirectly confirmed by measuring a step-wise increase of the membrane current upon laser illumination.

**Figure 2:**
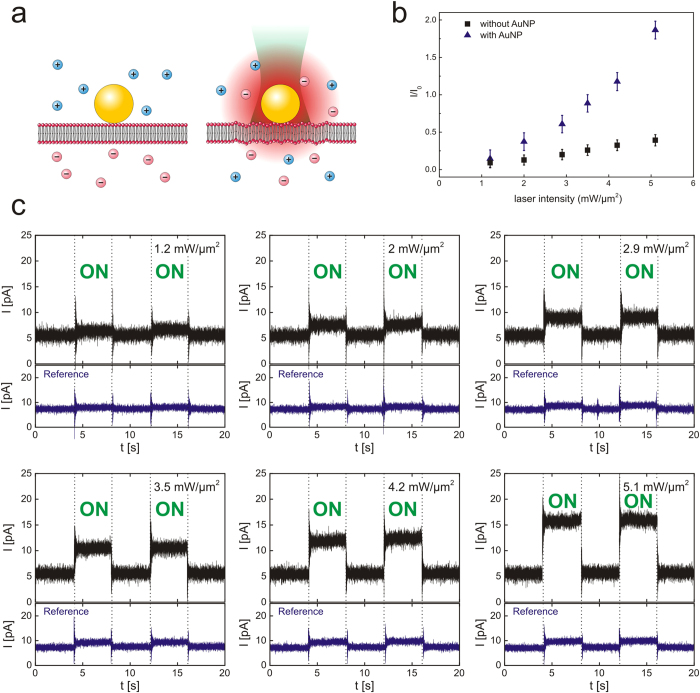
Heat dependent permeability of lipid membranes. (**a**) Gold nanoparticles are attached to a phospholipid membrane and illuminated with a laser (λ = 532 nm). Ions that drift along the field lines of an external electric field have a higher probability to cross the bilayer, resulting in a net current flow. (**b**) Laser dependent current measurements of a bilayer membrane with and without gold nanoparticles. The steps are normalized to the signal value before laser illumination in order to eliminate small variations in the bilayer conductance over time. (**c**) Detailed view on the laser power dependence of membrane currents. Current traces of a free standing lipid bilayer are measured after applying a constant voltage of +50 mV. The laser is switched on and off by hand. At the same time, the current signal shows reversible steps and recovers completely to the level before illumination after switching off the laser. The upper row (black) shows signals with a concentration of 1.09 × 10^9^ nanoparticles/ml in the recording solution. This particle concentration should result in the random deposition of approximately one nanoparticle in the observed time frame (supporting information, Fig. S1). As a reference, measurements without gold nanoparticles were performed for each laser power.

A schematic of the experimental procedure is shown in Fig. 2a. Without laser illumination, there is only a small current measured due to a small ion drift across the bilayer. As soon as the laser is turned on a sudden increase of the measured current is observed. The experimental results for the depicted heating experiment as a function of the laser power are shown in Fig. 2b. Heating of plasmonic nanoparticles happens on a very small time scale and a particle can heat and cool down within a few nanoseconds^41^. An elevated current at a constant level was measured over the whole time that the laser was switched on. When the laser was turned off, the current dropped to the initial value illustrating that the process is completely reversible and that the integrity of the membrane is not altered for the laser intensities that were used (Fig. 2c). The measurement became unstable, only if the laser power was set too high to induce membrane rupture and even the formation of vapor bubbles^42^. Experimentally that was observed by greatly fluctuating current levels and ultimately the breaking of the resistance seal.

We also found that the current strength depended on the laser power (Fig. 2b). The dependence of the laser power and the measured current was not linear, although one would expect a linear increase of the nanoparticle temperature for higher laser intensities. This observation can be explained since a continuous wave (*cw*) laser was used for particle heating. The temperature profile around a gold nanoparticle under *cw*-illumination drops as a function of 1/r (supporting information Fig. S2). At the same time, the bilayer area that is heated by a small particle increases by a quadratic order. The current dependence as a function of laser power thus shows a contribution of both effects.

We verified for each measurement that a significant current increase was only observed in presence of gold nano- particles. The comparison between measurements with and without the addition of particles is shown in Fig. 2c. A small membrane current was observed, possibly due to heating of the water, the glass support, or the membrane itself with the focused laser light. In any case, the current in the control measurements was always significantly smaller which emphasizes our initial argument that heating with nanoparticles is indeed much more efficient.

As already explained, the laser was focused to illuminate the whole area on top of the hole in the glass cover slip to make sure that it would heat any gold particle located on the membrane. This approach has the disadvantage that the gold nanoparticles cannot be located directly on the bilayer due to optical constraints. The data recordings shown in Fig. 2 do therefore not necessarily reveal the contribution of a single particle.

**Figure 3:**
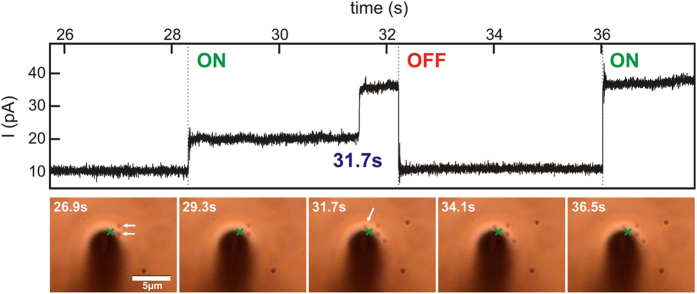
Real-time observation of patch-clamp measurements with simultaneous video microscopy. The area covered by the free-standing bilayer is shadowed by the cone going through the glass slide that is widening towards the bottom. In the beginning, two nanoparticles (white arrows) are visible near the focus of the laser beam (indicated by the green cross). After switching the laser on, the current signal goes up. At 31.7 s, a third particle is deposited at the position of the laser beam (31.7 s frame, white arrow). Correspondingly, the signal increases immediately by ~15 pA. After turning the laser off, the current recovers to the starting value, indicating that the bilayer seal is still intact and that the membrane is not ruptured. The increased current is maintained when the laser is switched on again (36 s) while the number of gold nanoparticles stays the same.

In order to confirm the impact of heating on bilayer permeability for individual particles we performed a slightly modified experiment shown in Fig. 3. Instead of focusing on the hole in the glass substrate, the laser beam was focused on gold nanoparticles that were located in the vicinity of the rim. Single gold nanoparticles are visible in brightfield mode in this configuration due to their high absorption cross-section (supporting information, Fig. S3). The approach to now illuminate individual gold particles in an area where the membrane is supported by the glass cover slip is, again, reasoned by the fact that the heat profile emerging around the nanoparticle can be larger than the nanoparticle itself due to the 1/r temperature distribution around the particle and the quadratic increase of the heated area as already explained earlier (supporting information, Fig. S2). Unlike before, we performed the experiment with high magnification and recorded simultaneously the current trace and a video as the experiment proceeds. The higher magnification objective defines a smaller focus of the laser beam (FWHM = 780 nm) and allows a better optical control of the experiment. As shown in Fig. 3, the center of the laser spot was located close to two gold particles. The laser was switched on and off, which was always accompanied by a stepwise change of the measured current signal. This indicates that the particles were still heated by the laser, although they were not located in the laser focus and the precise temperature of each particle could therefore not be determined. When the laser was switched on, a current increase of ~10 pA was observed. After some time, a third particle was deposited at the substrate and at the laser spot, likely induced by an optical printing mechanism due to optical forces acting on the particle as it was diffusing into the laser beam^43^. At the exact same moment of the attachment, a current increase of almost 15 pA was observed instantaneously that stems from the heating of this newly attached particle alone. It is important to note, that during the whole process, no irreversible damage to the bilayer is observed and without laser illumination, the current level dropped to its initial value. As discussed in Fig. 2, the measured current shows a strong dependence on the laser power, likely because a larger area of the membrane underneath the particle is heated due to heat dissipation for increasing particle temperatures.

**Figure 4:**
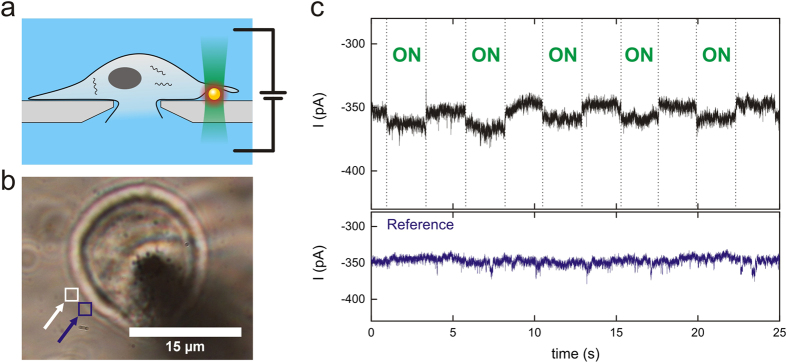
Current steps in membranes of living cells. (**a**) HEK293 cells are clamped in ‘whole-cell’ configuration. (**b**) A single gold nanoparticle located at the cell (indicated by a white arrow and a white box) is illuminated with a laser beam (λ = 532 nm). (**c**) A reversible increase of the ion current was observed when the laser was switched ‘on’ and ‘off’ (top panel). Please note that the patch-clamp measurements of cells that are shown here were performed with a negative (−60 mV) holding potential. The overall current increase that was measured upon laser irradiation has therefore also a negative sign. Similar to the bilayer recordings shown in Fig. 2, no current increase was observed in control measurements without a gold particle (‘Reference’ measurement, lower panel). The laser position for the reference measurement is indicated with a blue arrow and a blue box in (**b**). The laser power density for all measurements was set to 11.8 mW/μm^2^.

Finally, we performed a proof of principle experiment to illustrate that the heating from a single particle can also be employed to switch membrane permeability in the membrane of a living cell (Fig. 4). We performed patch-clamp recordings on HEK293 cells. The glass slide was first incubated with gold nanoparticles and some particles were randomly deposited on the glass surface. In the next step, a cell was patched such that the cell body was sitting on top of the particles. The laser was focused on a single particle to heat a local spot on the cell membrane. Similar to our recordings on synthetic membranes, we found that also on the native membrane currents can be switched on and off, remotely controlled by the laser beam. For the experiments on living cells, we cannot exclude that particle heating might also affect membrane channels and proteins that are present in the native environment. The reversibility of the membrane resistance over several cycles of laser illumination however suggests that no damage of the immediate environment around the particle has occurred.

Understanding the processes governing the temperature dependence of ion permeability of lipid bilayers requires a deeper view at the actual ion translocation mechanism. Picturing the bilayer as a simple rigid system, an ion would need to cross from a high (water) to a low (hydrocarbon) dielectric environment. This model falls short in explaining the real situation as it has been shown that simple energetic considerations already lead to a difference of several orders of magnitude between different ions and an overall lower conductivity compared to experimental observations^38^,^44^,^45^. In order to account for these discrepancies between theory and experiment, several models have been proposed that also involve the reorganization of lipid molecules. One of these model is based on the formation of transient pores and consequently the passage of ions through the resulting water column^46^. Experimentally, such pores can be induced by applying short electrical pulses (electroporation)^47^. The conductivity signature of transient pores, however, is very distinct. Usually there are two conductivity levels and the current fluctuates stochastically between the two levels, indicating random pore opening and closing, very similar to ion channel openings^48^. Currents that are observed due to membrane phase transitions or mediated by impurities also show this characteristic signature^49^. No random current jumps were observed in the experiments reported here. Instead, laser heating leads to a fully deterministic and reversible change of the current level.

Furthermore, it is important to consider that the temperature increase might also change the properties of the buffer solution. The Walden Rule gives a simple model for the relation between viscosity and conductivity of a buffer solution^50^. The viscosity of an aqueous solution decreases with temperature^51^ which then influences ionic conductivity. Indeed, Olapinski *et al*. have reported that a temperature dependence for patch-clamp experiments can be observed^52^. The electrical equivalent circuit of the measurement reported here can be described as a series of the resistance of the hole in the glass slide and the resistance of the bilayer membrane. Since the bilayer resistance is several orders of magnitude higher than the resistance of the hole, ion conductivity of the solution only plays a minor role in the case reported here.

In light of these arguments and our experimental observations, we instead propose a mechanism introduced by Wilson *et al*.^53^, where the charged ion itself perturbs the bilayer, to be the dominating process for the occurrence of ion translocation and the observation of membrane currents. This is based on the idea that an ion entering the hydrophilic headgroup region of a bilayer perturbs the membrane by pulling the headgroups inward. The resulting defect lowers the overall energy barrier for the ion to pass. Localized heating facilitates this process. This is in accordance with previous studies by other groups, where molecular dynamics simulations have shown that the required energy for ions to translocate is indeed depending on the temperature of the system^54^.

In conclusion, we have demonstrated that local heating can be applied to reversibly switch the permeability of lipid membranes. For this, we have taken advantage of the ability of gold nanoparticles to efficiently convert light into heat, creating small, localized heat sources. The whole process is monitored with brightfield microscopy and patch-clamp equipment. The increase in conductivity depends on the laser power which is in agreement with the theoretical temperature profile of a heated gold nanoparticle in water. Employing gold nanoparticles enables a rapid change of the temperature, allowing the decoupling of the different mechanisms proposed to increase the permeability of lipid bilayers. Overall, this approach of controlling biological systems and cellular function with high spatio-temporal resolution paves the way for future biomedical applications in the field of nanotheranostics and drug delivery.

## Materials and Methods

DPhPC (1,2-diphytanoyl-sn-glycero-3-phosphocholine), dissolved in chloroform is purchased from Avanti Polar Lipids. Sorbitol and Cholesterol are purchased from Sigma-Aldrich. Gold nanoparticles with a diameter of 80 nm (citrate stabilized) are from Nanopartz. Consumable plastic caps for the Nanion Port-A-Patch (NPC-1, 3–5 MΩ) are directly bought from Nanion.

Giant Unilamellar Vesicles (GUVs) are prepared by electroformation^55^. In short, lipids (DPhPC and Cholesterol at a ratio of 9:1) dissolved in Chloroform are spread on two parallel platinum wires in a Teflon chamber. After drying, Sorbitol solution (1 mol/l) is added, the chamber is heated to 50 °C and an alternating electric field is applied for 120 min (5 Hz, 3Vpp). Vesicles are stored at 4 °C until further use.

The HEK293 cell line, kindly gifted by Prof. Dr. Joachim Rädler, LMU Munich, was cultured in RPMI 1640 medium supplemented with 10% fetal bovine serum (FBS) and 2 mM L-glutamine. Cells were incubated at 37 °C (5% CO_2_) until 80–95% confluence before trypsinization.

Experiments were performed on a commercial setup for planar Patch Clamp (Nanion Port-A-Patch). A custom made holder for the microscope add-on was built in home and used in combination with an upright microscope (Zeiss Axio Scope.A1). A laser (λ = 532 nm, LaserQuantum) is coupled into the microscope and a 10× or 100× water immersion objective is used simultaneously for focusing the laser and for optical observation. Photos and videos are acquired with a Canon EOS 550 D camera.

For patch-clamp recordings on lipid membranes with the Nanion Port-A-Patch, the bilayer is formed over a micrometer-sized hole in a glass slide. The slide is incorporated into a consumable plastic cap. a droplet of buffer solution (50 mM KCl, 10 mM NaCl, 60 mM K-Fluoride, 20 mM EGTA, 10 mM HEPES, adjusted to pH 7.2 with KOH) is added to the inside (5 μl) and outside (10 μl) of the cap. Then 5 μl of solution containing vesicles is added to the top of the cap and suction is applied until a Gigaseal is formed (see supporting information, Fig. S4 for an SEM image of a dried sample after the experiment). Consequently currents are recorded with a Patch-Clamp amplifier (EPC 10 USB Single, HEKA Elektronik) and Ag/AgCl electrodes on both sides of the bilayer. Current is measured continuously at a bias of +50 mV and is sampled at 1 kHz. Patch-Clamp on cells is performed in whole-cell configuration at a holding potential of −60 mV. The same buffer solution as above is used as intracellular solution, the buffer on top of the cap is replaced with extracellular solution (140 mM NaCl, 4 mM KCl, 1 mM MgCl_2_, 2 mM CaCl_2_, 5 mM D-Glucose, 10 mM HEPES, adjusted to pH 7.4 with NaOH). Prior to the experiments, cells were harvested, centrifuged for 3 min at 1000 rpm and redispersed in extracellular recording solution. Whole cell configuration was obtained by standard protocols for the Port-A-Patch, provided by Nanion.

## Additional Information

**How to cite this article**: Urban, P. *et al*. Reversible control of current across lipid membranes by local heating. *Sci. Rep.* **6**, 22686; doi: 10.1038/srep22686 (2016).

## Supplementary Material

Supplementary Information
