# Supplementary material for: Reversible control of current across lipid membranes by local heating
Source: Sci Rep. 2016 Mar 4;6:22686. doi: 10.1038/srep22686 (PMC4778043; doi:10.1038/srep22686)
Supplement: Supplementary Information [file srep22686-s1.pdf]

Supporting Information

-

Reversible control of current across lipid membranes by local heating

Patrick Urban[1], Silke Kirchner[1], Christian Mühlbauer[1], Theobald Lohmüller[1,2], and Jochen Feldmann,[1,2]*

[1]Photonics and Optoelectronics Group, Department of Physics and Center for NanoScience (CeNS), LMU München, Amalienstraße 54, Munich, 80799, Germany

[2]Nanosystems Initiative Munich (NIM), Schellingstraße 4, 80539 Munich, Germany

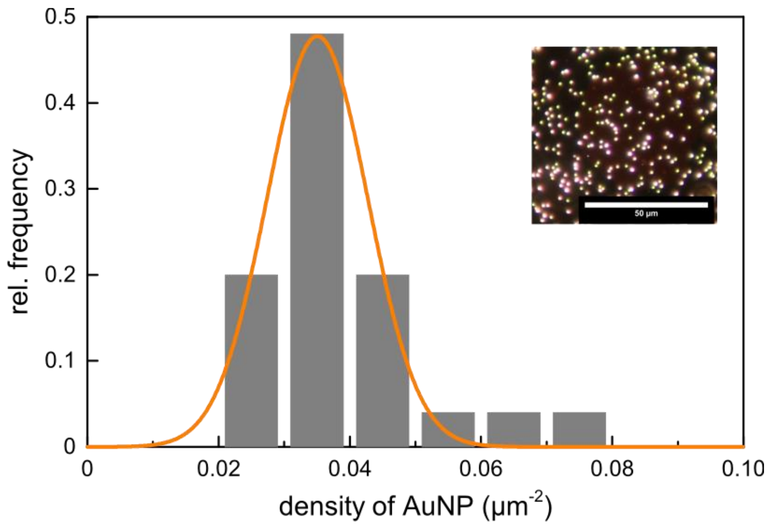

Figure S1: Density of gold nanoparticles: In order to estimate the density of gold nanoparticles adsorbed to the bilayer over time, the experimental conditions were reproduced with standard glass coverslides. A droplet of buffer solution was given on a glass slide. Vesicles with a small amount of fluorescing lipids were left to settle and rupture on the substrate. Gold nanoparticles were added to the same concentration used in the experiments. After a few minutes, dark-field microscope pictures of several areas were taken (example shown as inset). The number of particles per bilayer and the area of the ruptured vesicle were measured by dark-field microscopy. The Gaussian fit to the frequency (orange) yields a density of 0.35±0.07 particles per µm².

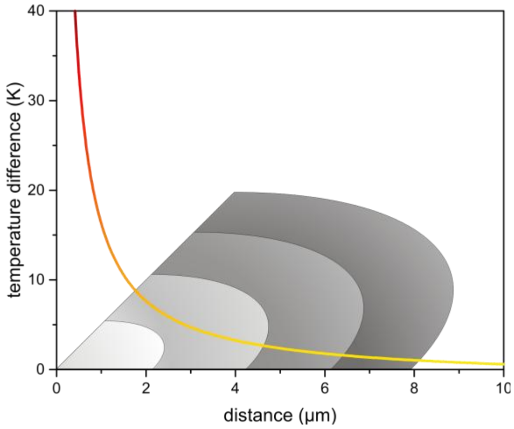

Figure S2: Simulated heat profile around a gold nanoparticle illuminated with a green, cw laser with a laser intensity of 7.3 mW/µm². The temperature decrease as a function of the radius of the heated area is sketched in different shades of grey. The radial distribution demonstrates a similar trend as the experimental data, indicating a relationship between temperature and the ion permeability.

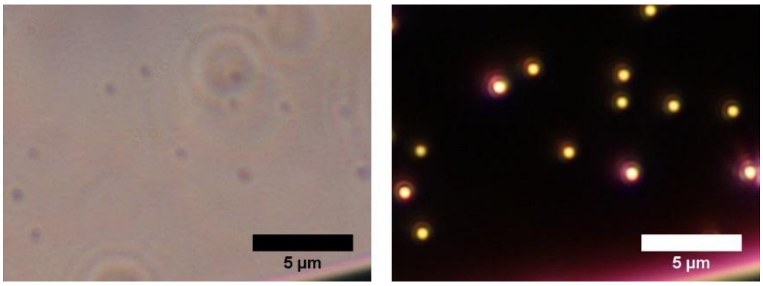

Figure S3: Gold nanoparticles on glass observed in bright-field (left) and in dark-field (right) mode. Dark-field microscopy is advantageous for optical imaging of single gold nanoparticles due to their high scattering cross section. The dark-field image shows a high contrast due to the dark background. The contrast for bright-field images is much lower, but single particles are still visible as darker spots.

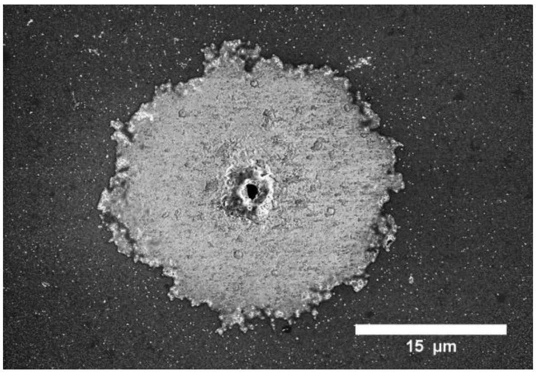

Figure S4: SEM image of a bilayer formed on the Patch-clamp chip. Before imaging, the sample was dried and sputtered with a thin layer of gold/palladium. Drying destroys the free standing bilayer membrane covering the hole in the glass slide and it becomes visible in the center of the image. Gold nanoparticles are visible as white spots.
